# Hemoglobinopathy screening in primary care in the Netherlands: exploring the problems and needs of patients and general practitioners

**DOI:** 10.1038/s41431-022-01156-0

**Published:** 2022-08-09

**Authors:** Margo E. van Vliet, Jean-Louis H. Kerkhoffs, Cornelis L. Harteveld, Elisa. J. F. Houwink

**Affiliations:** 1 Medical faculty, Leiden University Medical Center, postal zone V0-P, PO Box 9600, 2300RC Leiden, The Netherlands; 2 Department of Hematology, HAGA Hospital, Els Borst Eilersplein 275, 2545AA The Hague, The Netherlands; 3 Laboratory for Diagnostic Genome Analysis, Department of Clinical Genetics, Leiden University Medical Center, postal zone S6-P, PO Box 9600, 2300RC Leiden, The Netherlands; 4 Department of Public Health and Primary Care, Leiden University Medical Center, postal zone V0-P, PO Box 9600, 2300RC Leiden, The Netherlands

**Keywords:** Anaemia, Population screening

## Abstract

The prevalence of hemoglobinopathies in The Netherlands is increasing due to migration. Hemoglobinopathies are severe hereditary diseases. An informed reproductive choice by at-risk couples, such as pre-implantation diagnosis or termination of affected pregnancies, can be made if carriers are detected prior to conception. Using a qualitative design, the needs and wishes of patients, carriers and general practitioners were evaluated regarding carrier detection of hemoglobinopathies in primary care practice. 30 semi-structured interviews were established with 10 general practitioners, 10 patients and 10 carriers. The interviews were audio-recorded, transcribed verbatim and analysed using content analysis to identify recurring themes. Three themes were generated regarding carrier detection of hemoglobinopathies: (1) a need for more information about hemoglobinopathy, (2) a need for indications when to refer for analysis (carrier diagnostics) and (3) insight concerning organization and roles in care for hemoglobinopathy carriers and patients. These themes reflected a need to increase awareness of hemoglobinopathy, improve competences among general practitioners through better education and improvement of communication with patients and their unidentified family members. This study shows the scope of the problem and the critical need for action to improve informed reproductive decision making for the at-risk population.

## Introduction

Hemoglobinopathies (HBPs) are the most common monogenic autosomal-recessive diseases in humans. The most severe types are sickle cell disease and alpha/beta thalassemia major. Carriers are generally asymptomatic and unaware of their carrier status. Therefore, carrier couples are often unaware that each pregnancy they are facing a 25% genetic risk of having a child with HBP.

Genetic defects in the globin genes encoding the hemoglobin protein are the cause of HBPs. Thalassemia patients have expression defects of the globin genes leading to chronic hemolytic anemia, while sickle cell patients have a specific defect in the globin gene which may lead to polymerization of HbS in deoxygenated state and deformity of the erythrocytes known as sickle cells.. These expression and structural defects have severe effects on the patients [1]. Palliative treatment of HBPs is available, consisting of administration of hydroxyurea or blood transfusions. A hematopoietic stem cell transplantation is the only curative treatment, however compatible stem cell donors are not always available and the procedure can cause severe complications such as graft-versus-host disease as well as infections [2].

HBPs are frequently occurring in regions where malaria is or was endemic, as the heterozygous carrier status protects against death from malaria tropica in infancy [3]. Increasing migration on a global scale caused HBPs to become more common in regions non-endemic for malaria, such as North-America and Northern Europe. Nowadays in The Netherlands, around nine children are born with homozygote or compound heterozygote HBP mutations for every 10,000 migrant births [4].

In 2007 sickle cell disease and beta thalassemia were added to the so-called ‘heel prick’ test, a blood sample taken in the first week after birth [5]. However detecting beta thalassemia carriers is not feasible in the first week of life, because expression of the characteristically elevated hemoglobin A2 (HbA2) is still too low in the first months after birth. From 2007 about 800 HbS heterozygotes and 35–40 babies are detected yearly with sickle cell disease or beta-thalassemia major [6]. Antenatal screening or screening before conception, could be supplementary to the current neonatal screening. This will allow couples at-risk to make an informed reproductive choice for prenatal diagnostics, pre-implantation diagnostics or otherwise and prevent being unexpectedly confronted with a child with a severe disease [7].

Certain countries in Europe already offer antenatal screening for HBPs, for example the United Kingdom. The UK has a national antenatal screening program to test all pregnant women coming from high prevalence regions in early pregnancy [8]. In past studies, HBP screening in the Netherlands was predominantly regional, and involved screening pregnant women early in pregnancy in collaboration with midwives [7, 9]. In 2005, attitudes to antenatal HBP screening were evaluated among the three largest immigrant groups in the Netherlands, and 68% to 77% of participants expressed an interest in carrier testing. Furthermore, over 80% of a well-informed, multi-ethnic group of members of a patient organization preferred the option of selective termination in case of an affected foetus. This reflects the urgent need for carrier detection of HBP before conception [10].

Our previous study clearly showed the gap between estimated carrier prevalence and registration and thereby the pressing need for action [11]. It was recommended to perform a qualitative analysis to explore the causes of the low carrier registration by physicians in primary care. In the Netherlands patients are first seen by the general practitioner (GP). As this is common practice, the GP can fulfil an important role as gatekeeper being the first to suspect a carrier of HBP.

The aim of this study is to explore strategies for HBP screening in the preconception phase in high-risk patients, using qualitative analysis. The need for education and communication with patients and their unidentified family members are explored. This qualitative study discusses the detection of HBP early in primary care with patients, carriers and GPs.

## Methods

### Design

A qualitative study was designed using interviews of HBP patients, carriers and GPs because this enabled us to explore the knowledge about HBP and the need for education and communication in those areas perceived by these stakeholders.

### Participants

Data was collected based on semi-structured interviews of 10 HBP patients, 10 carriers and 10 GPs. The GPs were recruited by email via the HAGA hospital network and through the researcher’s personal networks. Patients and carriers were selected by purposive sampling of the HBP database at the HAGA hospital in The Hague and via the hemoglobinopathy patient organisation OSCAR, with the aim of increasing the diversity of characteristics and to obtain rich, relevant and diverse data. The participants were expected to provide complete and possibly complementary perspectives on the prevention of HBP through carrier screening in primary care. Potential participants were suggested by one of the authors (JK), hematologist in the HAGA hospital, an acknowledged centre of expertise in the field of hemoglobinopathies taking care of the majority of HBP patients in the region.

One interviewer (MV) conducted the semi-structured interviews in the Dutch language. All interviews were performed via a telephone call, which lasted approximately 15 minutes and were recorded. Due to the very busy schedules of the GPs and the COVID pandemic, we were not able to plan longer interviews. A translator was not needed because all participants were fluent in the Dutch language. Participants were interviewed only once. Before the interview the participants gave written informed consent and completed a questionnaire on population characteristics (see Appendix 1, 2). The participants were informed that they could withdraw from the study at any time and that there were no right or wrong answers.

### Interview guide

The interview used pre-set open-ended questions developed by the multi-disciplinary research team (see Appendix 3–8). The questions covered the following patient and carrier-related topics: personal experience of HBP, influence of HBP on decision making and the role of the GP in HBP carrier detection. Questions meant for GPs concerned knowledge of symptoms, disease severity and genetic consequences of HBP, experience with HBP patients, collaboration with other healthcare workers regarding HBP and the role of the GP in HBP carrier detection.

### Data analysis

Interviews were performed between July and December 2020. All interviews were audio recorded and transcribed verbatim by the interviewer using the software program ATLAS.ti. In order to identify the themes emerging from the interview transcripts of the GPs, patients and carriers, it was concluded this research should focus on identifying themes within the understanding of the participants. This provides the researcher with scope for further investigation of this subject. Therefore, we concluded that thematic content analysis was the most appropriate method. The authors of this study take a position acknowledging our aim to blend the individual experiences of the participants and the meanings they connect to them. Nonetheless, we also prefer to contemplate the effect of the wider social context on these meanings. Braun and Clarke previously specified such an attitude as “contextualism,”, which is placed between essentialism and constructionism [12, 13]. Pre-set questions were used in our interviews. After every round of interviews, the pre-set questions were reviewed in a peer debriefing session if the pre-set questions were still adequate and fit the research question. One round consisted of 4 interviews All transcripts were read to get familiarized with the data. The dataset is deductively coded. Both semantic and latent codes were used. Afterwards the initial themes were generated. The initial themes were reviewed and redefined following a structure of thematic analysis named by Braun and Clarke. All transcripts were independently coded by two researchers (MV, EH) [14], and differences in coding were discussed to enhance the display of the multiple various views of the participants.

## Results

All participating GPs acknowledged deficiencies in their knowledge concerning HBP and expressed a wish for additional education. In addition, many GPs were unaware of the different care trajectories of HBP patients or carriers and therefore the way potential HBP patients and carriers were approached differed considerably. Interestingly, no significant relationship was found between the number of people with a migration background present in a GP-practice and the knowledge and views of the GPs. The characteristics of patients, carriers and GPs are presented in Tables 1–3. Three major requirements were raised: [1] more information about HBP [2], clear indications for referral or more research and [3] a better understanding of organisation and roles in HBP care. In the following paragraphs the themes are presented in depth.

**Table 1:** The characteristics of the patients.

No.	Age (years)	Gender	Level of education	Ancestry	Type hemoglobinopathy
**1**	51	Male	HBO	Antilles	Sickle cell disease
**2**	30	Male	HBO	Dominican Republic/Antilles	Sickle cell disease
**3**	40	Male	MBO	Surinam	Sickle cell disease
**4**	46	Female	MBO	Turkey	Beta-thalassemia
**5**	74	Female	HBO	Surinam	Sickle cell disease
**6**	38	Female	HBO	India	Sickle cell disease
**7**	42	Male	MBO	Antilles	Sickle cell disease
**8**	23	Female	WO	Afghanistan	Beta-thalassemia
**9**	26	Female	MBO	Antilles	Sickle cell disease
**10**	23	Male	MBO	Antilles/Venezuela	Sickle cell disease

**Table 2:** The characteristics of the carriers.

No.	Age (years)	Gender	Level of education	Ancestry	Type of hemoglobinopathy carriership
**1**	29	Female	MBO	Netherlands/ Surinam	Sickle cell disease
**2**	45	Female	HBO	Netherlands/ Surinam	Sickle cell disease
**3**	46	Female	HBO	Netherlands/Indonesia	Beta-thalassemia
**4**	34	Female	HBO	Netherlands/Antilles	Sickle cell disease
**5**	34	Female	WO	Netherlands	Beta-thalassemia
**6**	19	Female	WO	Netherlands	Beta-thalassemia
**7**	52	Female	WO	Netherlands	Beta-thalassemia
**8**	40	Female	WO	Netherlands/Surinam	Sickle cell disease
**9**	67	Female	WO	Netherlands	Beta-thalassemia
**10**	49	Female	MBO	Netherlands	Beta-thalassemia

**Table 3:** The characteristics of the general practitioners.

No.	Age (years)	Gender	Work experience (years)	Type of GP	Inhabitants with a migration background in city of practice (%)
**1**	54	Female	24	GP partner	32.9
**2**	62	Male	30	GP partner	22.0
**3**	32	Female	2	Sessional GP	51.6
**4**	40	Female	10	Sessional GP	34.0
**5**	42	Female	14	Sessional GP	31.4 and 22.0
**6**	61	Male	31	GP partner	22.0
**7**	41	Male	6	Sessional GP	38.8
**8**	45	Male	15	GP partner	10.7
**9**	60	Female	32	Sessional GP	17.8
**10**	35	Female	5	Sessional GP	19.7 and 34.7

### The need for better knowledge of HBP

GPs recognized their deficiencies in knowledge regarding HBP, since they often had little or no clinical experience of the disease. Insufficient knowledge was identified concerning prevalence, heredity, severity and treatment options for HBPs. The severity was commonly underestimated, especially by GPs with no clinical experience with HBP, and one GP stated (woman, 40 years): *‘If a patient really has the disease rather than the trait, I think it will have a major impact on their life. As I always refer them, I know theoretically what it means to the patient but not in actual daily practice.’*

GPs with more clinical experience were better able to estimate the severity of HBPs. An example were two GPs who had previously worked in Africa, one of whom said the following (male, 61 years): *‘It is not so bad for carriers. Although as I saw in Africa, if you really have the disease, it is horrible […]. These were seriously ill patients and always having problems.’*

GPs had little confidence in their own knowledge and mentioned the need for more information before they would feel able to counsel a patient in relation to HBP. One GP answered a question concerning HBP counselling (female, 42 years): *‘At the moment I am absolutely not able to do it, and although I know you can offer embryo selection, I could not provide adequate counselling right now.’*

GPs expressed a wish for hands-on information about HBP, and there appeared to be a general demand for easily accessible information sources, such as websites, that provide brief, understandable information that can be used when communicating with their patients in daily practice. They also mentioned that a website should be maintained by qualified specialists. Nevertheless, interviewed GPs were unfamiliar with existing websites that provide information on HBP and other genetic diseases, such as “GPandgenetics” (in Dutch huisartsengenetica.nl) and hbpinfo.nl. Other suggestions for sources of information were review articles, flyers, refresher courses and flowcharts. Besides the need for more information for doctors themselves, the GPs also noted the lack of reliable information for patients other than in the English language, but also in mother tongue.

Patients and carriers also recognized a need for better knowledge of HBP in primary care and felt that they were losing trust in their GP because he or she lacked the knowledge to help them. One sickle cell disease patient said (male, 40 years): *‘Generally, if I have complaints I do not go to the GP as he only tells me to go to the hospital emergency department. So, I just skip the GP and call the hospital directly.’*

However, despite the common belief that carriers do not develop complaints related to their hemoglobinopathy mutation, almost all interviewed carriers reported complaints that they attribute to being a carrier. They regularly experienced a lack of empathy from their GP, as most GPs just send them home with the message that their complaints cannot be explained by HBP trait.

Patients and carriers also expressed a need for more information concerning the hereditary implications of the disease. Most interviewed patients and carriers were uncertain about the hereditary aspects of the disease and for example did not know which family members might be affected. Patients and carriers also expressed a wish for support from their GP when informing their family members. Even though hemoglobinopathy was not a taboo in most patient and carrier families, discussing the importance of the disease and of being a carrier of the disease seemed to be hampered by a lack of reliable and understandable information. Especially carriers expressed little urge to inform family members.

### The need for indications when to refer or perform diagnostics

GPs expressed a need for clear indications for referral and carrier diagnostics. Current approaches vary widely between GPs, with some performing diagnostics themselves, while others immediately refer to a center of expertise when they suspect HBP. Many GPs are unfamiliar with the characteristics of HBP and therefore uncertain when to perform diagnostics to detect HBP. As previously mentioned, there is an urgent need for clear information aiming at GPs that will help them decide when to perform diagnostics themselves and when to refer the patient to a Center of Expertise.

Patients and carriers both felt a need for clear procedures for the GP. All carriers interviewed were diagnosed incidentally, sometimes after years of ineffective iron therapy. Both patients and carriers reported that their GP was unaware of the importance of testing their partner and had to convince the GP of its importance themselves. The unawareness and late diagnosis potentially deprive the carriers from an informed reproductive choice, because sometimes they already had children before the diagnosis and the trait was already transmitted. All patients and carriers interviewed expressed a wish for partner testing and stated that they would recommend it to others. One beta-thalassemia carrier said (female, 34 years): *‘In our last pregnancy we discovered that a blood count alone is not enough to exclude a diagnosis of hemoglobinopathy; people with a normal blood count can still be a carrier. I know this now, but it would have been nice if I had known this before my pregnancies.’*

### Insight into organization and roles in hemoglobinopathy care

Most GPs had little understanding of the organization of HBP care, and thus referred their patients and carriers to a variety of other doctors including clinical geneticists, haematologists and clinical chemists. This variation reveals the current confusion regarding appropriate clinical trajectories for HBP patients and carriers. It is important for HBP screening that GPs have knowledge about the organization of HBP care, so they know the consequences of the disease and the proper way to refer for genetic counselling if a carrier couple is discovered. HBP care is an unfamiliar area for many GPs, as typified by the answer of one GP concerning HBP care in the Netherlands (male, 40 years): *‘No, I do not know where I should go [for HBP care]. It is totally unclear. It is an unfamiliar disease. Sometimes you see it in the list of problems, but you have little to do with it.’*

GPs were also uncertain regarding their own role in the screening of HBP carriers and do not consider HBP screening their task, while others were aware of this role, although they experienced difficulties due to a lack of time and knowledge. Most GPs who saw a role for themselves in screening was argued it was mainly signalling the problem. Once HBP is detected or suspected, they would rather refer to a specialist. Nonetheless, GPs also admitted that they need more knowledge even to complete this signalling task adequately. The information requested most ed involved prevalence per ethnicity and hereditary risks. One GP mentioned that roles in HBP carrier detection may differ between cities depending on the urban population (female, 32 years): *‘I think it really differs depending on your patient population, because if you have few patients with a migration background you will almost never see it [HBP]. In that case I think there is no role for the general practitioner.’*

Confusion was also noted regarding the ‘heel prick’. This blood test on all neonates born in The Netherlands is designed to detect a subset of genetic diseases including sickle cell disease, sickle cell trait and beta thalassemia major. Some GPs thought that screening at birth was sufficient, while others acknowledged that those who arrive in the Netherlands later should also be identified by the GP. As carrier detection is not the primary aim of heel-prick screening, it is important that GPs realize that alpha- or beta-thalassemia trait as well as carriers of common Hb variants are not reported. The latter are detected by HPLC but not reported in the heel-prick screening in The Netherlands for clerical reasons.

### Suggestions for strategies to perform HBP screening

The following general considerations were extracted from the interviews held: GPs expressed a desire for more information about HBP and genetics in general before they can adequately recognize and diagnose HBP and carriers of HBP. Patients and carriers also mentioned this issue because they often feel unseen and misunderstood. Patients and carriers expressed a need for clear information about the consequences of being a carrier and information about the disease to help them informing their families.

The GPs mentioned lack of experience with HBP as a reason to consult or refer to a specialist. The GPs would also like better insight into the organisation of HBP care and clear referral indications. This wish was also expressed by patients and carriers. Finally, strategies were suggested to improve HBP care by asking experienced GPs to take on the role of the expert GP and guide therapy. However, the timely recognition of high risk HBP patients or carriers should remain part of the gatekeepers role of all GPs as far as genetics, care and prevention are concerned. Other detailed strategies suggested to effectively incorporate HBP care into general practice, are stated in Table 4.

**Table 4:** The strategies to increase awareness for and knowledge about hemoglobinopathies.

Strategies	Details
E-health/online flowchart	A decision tree or decision support system should help GPs and midwives with busy schedules to quickly find when and how to test for carriership of HBP
E-learning/blended learning on HBP	Younger participants were in favour
Lectures/ overview articles	Preferred by older participants
Workshops	Studying by discussing clinical cases with colleagues. Basic genetic knowledge and skills about diagnostics, statistics and medical ethical topics, could be included.
Expert GP or nurse	One GP or nurse per neighbourhood specializes in HBP care and advices all other GP’s.

## Discussion

In this study the HBP-related preferences and experiences in primary care of HBP patients, HBP carriers and GPs were evaluated. Our previous article described the massive underdiagnosing of HBP in the region of The Hague**. Underdiagnosing HBP trait can be partly explained by a lack of awareness and knowledge amongst GPs. As mentioned previously, patients and carriers expressed a need for clear information, a wish that was also expressed in 2011 by focus groups of GPs, midwives and multidisciplinary groups. In that study GPs felt a more urgent need for education than the midwives, while the multidisciplinary group noted that both midwives and GPs lacked sufficient knowledge of genetics [15].

Currently patients and carriers often do not understand the GPs role and responsibilities in their disease. A survey-based study in the United States revealed a lack of confidence in GPs regarding all aspects of care for sickle cell disease patients and carriers. Experience with the disease was associated with increased confidence, as this study also showed in the case of GPs who had worked in Africa previously and had treated patients with sickle cell disease. Whiteman et al. suggests that it could be beneficial to select a group of motivated and interested primary care providers to specialize in primary care for sickle cell disease. They could assist other primary care providers or take over therapy [16].

### Strengths and limitations

This study highlights the problems concerning HBP in primary care from three viewpoints, those of the GP, the carrier and the patient. The outcome of the three interviewed groups were in concordance, which strengthens the results. Furthermore, purposeful sampling yielded a heterogeneous but representative group of GPs and patients reflecting the diverse, multi-ethnic population of the Netherlands. The analysis of qualitative data was performed by two researchers independently. After each cycle of interviews, the results were discussed until data saturation was reached. This iterative cycle improved objectivity and reliability.

Although data saturation was reached, the study is based on the opinions of a relatively small group of patient and GPs. Clearly, this may not be representative of all Dutch patients and GPs. The GPs interviewed were mainly working in The Hague, who are more prone to be confronted with HBP patients. However, we deliberately tried to include participants from a variety of backgrounds, for example both city and rural GPs, to achieve input on broader HBP-related developments in primary care. However, it remains to be seen whether our findings have relevance beyond the Dutch healthcare system, since the sample might reflect this specific healthcare system. Furthermore, the interviews last about 15 minutes due to the very busy schedules of the GPs and the COVID pandemic. This could raise questions about the quantity of information. However, this is a focused study on several themes, we did not intend to explore the whole topic of hemoglobinopathies. Another limitation is a possible bias due to self-selection because of purposive sampling. For example, most of the interviewed carriers were female and recruited with the help of the patient organisation OSCAR-Nederland, and it seems likely that carriers belonging to a patient organisation may be experiencing greater than average problems due to HBP trait.

### Future implications

Already in 1998 researchers were thinking about a screening program in The Netherlands, however this was felt to be inappropriate, because of a low number of diagnosed children, a poor understanding of HBP in the at-risk population and difficulties concerning the best moment for screening or prevention [17]. However, since 1998 migration increases in The Netherlands and nowadays 24.4% of the total Dutch population has a migration background [18]. The World Health Organization (WHO) published in 2006 two resolutions on HBP [19] and encouraged a screening program when there are more than 20 affected births per year. In The Netherlands 42 children are diagnosed by the heel prick screening in 2018, thus according to the WHO a screening program is warranted. The heel prick screening is not sufficient as a screening program as the consequences cannot be averted, because of the detection after birth.

To ensure better healthcare for people with a high-risk ancestry, a practical screening program for HBP trait is needed that aims for autonomous reproductive decision-making. The first step is informing both healthcare providers and patients. Screening for HBP can be performed by several different healthcare providers, such as the midwife or the GP. However, by the time the midwife detects a carrier couple through a pregnant woman, pregnancy of a possibly affected foetus is already a fact and drastic decisions have to be discussed during genetic counselling.

**Fig. 1:**
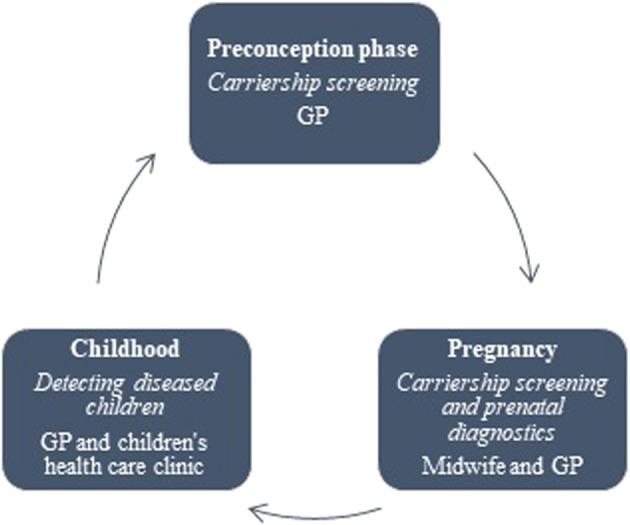
Model of roles in primary care. The roles of different primary care professionals in carrier detection.

In our opinion, it would be helpful to assign carrier screening to the GP in collaboration with the midwife, the child healthcare clinic and the genetic counsellor. Each healthcare worker can support their patients´ healthcare needs and contribute to the pre-conceptional or antenatal screening of hemoglobinopathies. Figure 1 illustrates the roles of healthcare workers. All the healthcare workers in the figure can refer a couple at risk to a genetic counsellor to discuss the options suitable for their situation. The GP is involved at every step, although most GPs interviewed in our study were unaware of this important role. We also found that GPs feel a need for more information, better criteria for referral and better insight in the organisation of HBP care. Hence, rather than just abandoning this task, preliminary work is needed. GPs need to know how to access reliable and hands-on information that boosts awareness. This can be achieved by, for example, promoting reliable websites such as “GPand genetics” or by providing e-learning. Diniz et al. found that a single instance of distance education (e-learning) already significantly improves understanding of sickle cell disease amongst healthcare providers [20]. Combining distance and regular education has been identified as one of the most effective ways to teach healthcare workers about genetics [21, 22]. These information resources need to provide clear criteria for carrier diagnostics and when to refer to a clinical geneticist, for instance by developing a flowchart (see appendix 9).

Another option to improve the awareness of HBP is registration of ethnicity in the GP electronic healthcare record (EHR). This would allow the system to automatically provide a reminder to ask about the patient’s family history and wishes regarding pregnancy, thus helping to assess whether carrier diagnostics is necessary. However, registration of ethnicity is controversial due to the risk or perceived risk of discrimination. Further societal discussion, involving healthcare providers and patients, is needed to find a way to include ethnicity as a risk factor for various diseases. By law (data protection law, article 18) registration of race is only allowed in The Netherlands for the purposes of positive discrimination, providing an advantage for the registered group. In the case of registration for HBP carrier detection, this seems to fall within the criteria of positive discrimination as this would allow the population at risk a fair chance to make important reproductive choices. It is debatable whether registration of ethnicity is the only way to provide this advantage, since heel prick screening is also available in The Netherlands for all newborn. However, as already mentioned, a considerable proportion of the at-risk population migrating into the Netherlands at a later age never underwent heel prick screening. Whether the registration of ethnicity is feasible and permissible by law deserves further examination by experts in health law.

The findings of this study are also applicable to other countries in Northern Europe with comparable immigration numbers. Aguilar Martinez et al. rated awareness in Belgium and The Netherlands as low, and Germany as very low. Germany even lacks a neonatal or antenatal screening program. The United Kingdom is an example of how other Northern European countries can do better, as the UK has national registration, a neonatal and antenatal screening program and a relatively high awareness [23].

In conclusion, carrier detection for HBP is necessary to ensure the health of populations from high-risk countries. The problem is still underestimated by GPs and opportunities to detect patients and carriers remain unused. Patients, carriers and GPs all agree that detecting carriers is necessary to improve reproductive decision making. However, GPs still lack knowledge and information to the detriment of patients and carriers. Possible screening programs and educational opportunities for GPs need to be investigated, with the aim to develop a practical and successful screening strategy. This study clearly shows the critical need for action to ensure optimal healthcare among a large at-risk population.

## Supplementary information

Supplement 1

## Data Availability

The datasets generated and analysed during the current study are available from the corresponding author on reasonable request with future collaboration as a main goal.
