# Supplementary material for: Hemoglobinopathy screening in primary care in the Netherlands: exploring the problems and needs of patients and general practitioners
Source: Eur J Hum Genet. 2022 Aug 9;31(4):417–23. doi: 10.1038/s41431-022-01156-0 (PMC10133269; doi:10.1038/s41431-022-01156-0)

**Supplement 1**

**Appendices**

*Appendix 1: Characteristics questionnaire GP*

Age: … years old

Gender: Man/Woman

Type of GP: sessional GP/GP in training/GP/other

Years of clinical experience:

Place of current employment: …………

*Appendix 2: Characteristics questionnaire patient*

Age: … years old

Gender: Man/Woman

Level of education: no/primary education/ lower secondary education/high school/higher professional education/university

Country of birth:

Country of birth mother:

Country of birth father:

Kind of disease: sickle cell disease/beta thalassemia

*Appendix 3: Questions for general practitioners, in English*

*Interviewer: this interview is about HBP and the prevention of HBP. When I refer to HBP in this interview I mean sickle cell disease and thalassemia major. Sickle cell disease and thalassemia are two different diseases, although they belong to the same etiological category.*

1. Can you describe sickle cell disease and beta-thalassemia and how often do you see patients with these diseases in your practice?
2. How severe do you think living with HBP is?
3. What are the common problems faced by patients with HBP and how do you deal with those problems as a general practitioner?
4. Is treatment of HBP possible? If yes, describe possible treatments?
5. Why and when would you refer a patient with a suspicion of HBP?
6. Who would you consult concerning HBP?
7. Thinking about prevention of HBP, which healthcare professional do you think is the best equipped to deal with HBP? Do you think the general practitioner has a role in diagnosing or treating a HBP patient?
8. What information do you need as a general practitioner to counsel a HBP patient and how would you like to receive this information?
9. Do you think the websites ‘huisartsengenetica.nl’ and ‘hbpinfo.nl’ play a role as an information resource? Which information do you need?

*Appendix 4: Questions general practitioner in Dutch*

*Interviewer: dit interview zal gaan over hemoglobinopathieën en eventuele preventie hiervan. Onder deze term versta ik in dit interview sikkelcelziekte en thalassemie major, dit zijn uiteraard twee verschillende ziekten, maar zij vallen in dezelfde etiologische categorie.*

1. Wat zijn sikkelcelziekte en thalassemie voor ziekten en hoe vaak komt u dit tegen in uw praktijk?
2. Hoe ernstig denkt u dat sikkelcelziekte en thalassemie zijn?
3. Welke problemen ziet u meestal opkomen bij uw patiënten of dragers met HBP en hoe vindt u dat de huisarts hiermee om zou kunnen gaan?
4. Denkt u dat behandeling mogelijk is voor HBP en welke behandelingen bestaan er dan voor HBP?
5. Waarom en wanneer verwijzen bij vermoeden risico op HBP?
6. Met wie zou u overleggen over HBP?
7. Als u denkt aan preventie van HBP, wie zou dat het beste kunnen doen? Denkt u dat er een rol is voor de huisarts om HBP te diagnosticeren en te behandelen?
8. Wat heb je als huisarts nodig aan informatie om de patiënt te counselen in preventie van HBP en hoe wilt u deze informatie dan krijgen?
9. Ziet u ook nog een rol voor de website huisartsengenetica.nl en hbpinfo.nl en welke informatie zou hierop moeten staan volgens u?

*Appendix 5: Questions for patients, in English*

1. What does having HBP mean to you?
2. How did you/your parents find out you have a HBP?
3. What kind of effect does HBP have on your daily life? How often are you ill or absent from work or school? Do you experience limitations in physical activity?
4. Do you think you live a different life compared to not having a HBP? Have you made different choices in life?
5. What do you expect from your general practitioner in terms of recognizing HBP complaints?
6. What does having a pain crisis mean for you?
7. Which treatments have you had for your HBP and what were they? Did you feel better afterwards and how often do you need treatment?
8. When you think about a HBP treatment, which treatment do you think of?
9. Do you think HBP prevention before pregnancy is sensible? Would you advise others to get tested if they might be a carrier of a HBP gene?
10. How do you see your future with HBP?

*Appendix 6: Questions patients in Dutch*

1. Wat betekent het voor u om deze aandoening te hebben?
2. Hoe bent u/uw ouders er achter gekomen dat u HBP hebt?
3. Welk effect heeft HBP op uw dagelijks leven? Hoe vaak bent u ziek of afwezig van werk of school? Zijn er beperkingen in fysieke activiteiten?
4. Betekent het hebben van HBP dat u een ander leven hebt dan wanneer u het niet had gehad? Heeft u andere keuzes in uw leven gemaakt?
5. Wat verwacht u van uw huisarts als het gaat om het herkennen van klachten die mogelijk bij HBP passen?
6. Wat betekent een pijncrisis voor u?
7. Wat voor behandeling voor HBP heeft u gehad en hoe zag dit eruit? Voelde u zich beter erna en hoe vaak moest de behandeling herhaald worden?
8. Als u denkt aan een behandeling voor HBP, welke behandeling ziet u dan voor u?
9. Denkt u dat preventie van HBP voor de zwangerschap zinvol is? Zou u andere adviseren zichzelf te laten testen of zij drager zijn van HBP? Waarom wel of niet?
10. Hoe ziet uw toekomst eruit met HBP?

*Appendix 7: Questions for carriers, in English*

1. How and when did you discover you are a HBP carrier?
2. Does being a carrier affect your daily life?
3. Has being a carrier influenced your life decisions?
4. How did your family/friends react to the news that you are a HBP carrier?
5. Which information sources do you feel were the best concerning HBP?
6. If you have a partner: would you advise your partner to test for carriership before embarking on pregnancy? If you already have children: did your partner get tested?
7. As a carrier, which information would you like to receive before pregnancy? Who would preferably provide this information?
8. Do you think HBP prevention before pregnancy is sensible? Would you advise others to get tested if they might be a carrier of a HBP gene?

*Appendix 8: Questions carriers in Dutch*

1. Hoe en wanneer heeft u ontdekt dat u drager bent van een hemoglobinopathie?
2. Merkt u iets van dat u drager bent in uw dagelijks leven?
3. Heeft het dragerschap invloed gehad op keuzes in uw leven?
4. Hoe hebben uw familie/vrienden gereageerd op het feit dat u drager bent?
5. Aan welke informatiebron heeft u het meeste gehad om te weten te komen wat HBP inhoudt?
6. Indien u een partner heeft: zou u willen dat uw partner zich laten testen op dragerschap voor u eventueel aan kinderen begint? Of indien u al kinderen heeft, heeft u dit gedaan?
7. Wat zou u prettig vinden om te weten als drager voor u aan kinderen begint? En van wie zou u deze informatie het best kunnen ontvangen?
8. Denkt u dat preventie van HBP voor de zwangerschap zinvol is? Zou u andere adviseren zichzelf te laten testen of zij drager zijn van HBP? Waarom wel of niet?

*Appendix 9: flowchart*


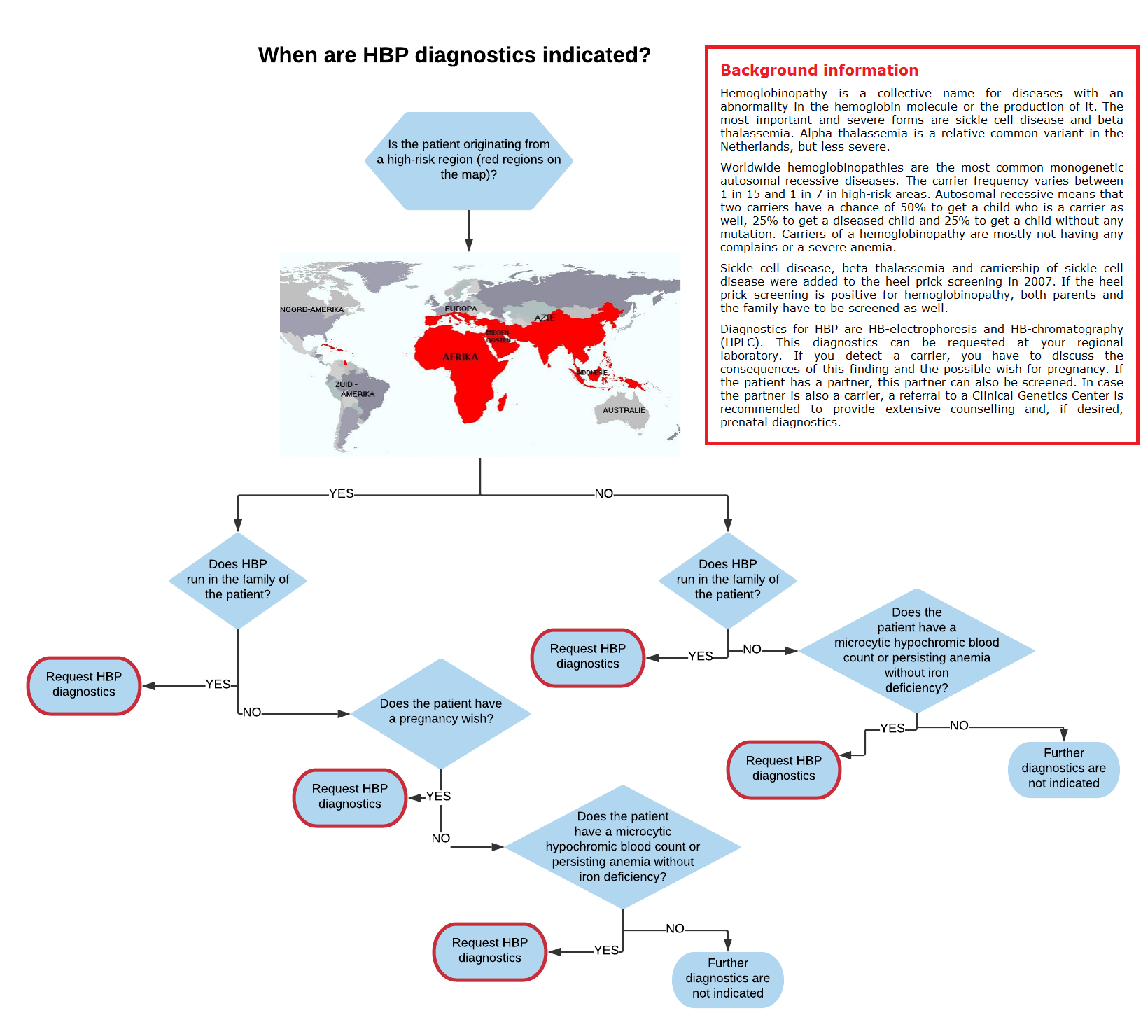

Supplement: Supplementary file 1 — Supplement 1 [file 41431_2022_1156_MOESM1_ESM.docx]
